# In Achilles Tendinopathy the Symptomatic Tendon Differs from the Asymptomatic Tendon While Exercise Therapy Has Little Effect on Asymmetries—An Ancillary Analysis of Data from a Controlled Clinical Trial

**DOI:** 10.3390/jcm12031102

**Published:** 2023-01-31

**Authors:** Goran Radovanović, Sebastian Bohm, Adamantios Arampatzis, Kirsten Legerlotz

**Affiliations:** 1 Movement Biomechanics, Institute of Sports Sciences, Humboldt-Universität zu Berlin, 10099 Berlin, Germany; 2 Department Performance, Neuroscience, Therapy and Health, Faculty of Health Sciences, Medical School Hamburg—University of Applied Sciences and Medical University, 20457 Hamburg, Germany; 3 Institute of Interdisciplinary Exercise Science and Sports Medicine, Faculty of Health Sciences, Medical School Hamburg—University of Applied Sciences and Medical University, 20457 Hamburg, Germany; 4 Department of Training and Movement Sciences, Humboldt-Universität zu Berlin, 10099 Berlin, Germany

**Keywords:** Achilles tendinopathy, inter-limb asymmetry, heavy slow resistance training, eccentric exercise, high-load, physiotherapy, tendon adaptation

## Abstract

Background: As inter-limb asymmetries can be associated with higher injury risk, we aimed to investigate their role in Achilles tendinopathy patients. Methods: In Achilles tendinopathy patients (n = 41), we assessed inter-limb asymmetries of mechanical, material, and morphological musculoskeletal properties and function and how those were affected by 12 weeks of exercise intervention (high-load protocol, n = 13; Alfredson protocol, n = 11). Moreover, we assessed whether asymmetry reductions correlated with improved Patient-Reported Outcomes (VISA-A score). Results: At baseline, tendinopathic tendons demonstrated lower tendon force (*p* = 0.017), lower tendon stress (*p* < 0.0001), larger tendon cross-sectional area (CSA) (*p* < 0.001), and increased intratendinous (*p* = 0.042) and tendon overall (*p* = 0.021) vascularization. For the high-load group, PRE-to-POST asymmetry comparisons revealed an asymmetry increase for the counter-movement jump (CMJ) (*p* = 0.034) and PRE-to-POST VISA-A score improvements correlated with CSA asymmetry reductions (*p* = 0.024). Within the Alfredson group, PRE-to-POST VISA-A score improvements correlated with CMJ asymmetry reductions (*p* = 0.044) and tendon stiffness asymmetry increases (*p* = 0.037). POST-to-POST in-between group comparisons revealed lower asymmetry in the high-load group for tendon elongation (*p* = 0.021) and tendon strain (*p* = 0.026). Conclusions: The tendinopathic limb differs from the asymptomatic limb while therapeutic exercise interventions have little effect on asymmetries. Asymmetry reductions are not necessarily associated with tendon health improvements.

## 1. Introduction

Achilles tendinopathy is one of the most common injuries of the lower extremities. It is characterized by alterations of tendon microstructure and is associated with pain, swelling, and a loss of function [1,2]. Lifetime prevalence in the general population is reported to be 5.9% while it is even higher in athletes [3]. Although multifactorial in origin, tendon mechanical overload is regarded as the main contributing factor [4,5,6].

Inter-limb asymmetry may contribute to either the onset or the persistence of musculoskeletal injury. Previous research reported that an inter-limb asymmetry greater than 10–15% is associated with increased injury risk and reduced performance [7,8], whereas inter-limb asymmetries lower than 10% for strength or function are considered as threshold allowing return-to-sport [9]. In the context of tendinopathies, greater asymmetry has been detected in patellar tendinopathy patients in comparison to controls while this was accompanied by a higher lower limb musculoskeletal injury risk in general in those patients [10]. Inter-limb strength asymmetry may lead to non-symmetric loading, which may in turn increase the risk of overloading [11], unequal force absorption, and reduced stability [12] due to alterations in motor behavior [13]. Indeed, strength asymmetry has been reported to be a risk factor for lower limb tendinopathy [14], suggesting a negative impact of pronounced inter-limb differences. In agreement, two current reviews demonstrated evidence for strength deficits of the tendinopathic side in Achilles tendinopathy patients [15,16]. Furthermore, higher tendon strain and reduced tendon stiffness for the tendinopathic Achilles tendon [17] have been reported. Increased tendon thickness and hypervascularization was reported in symptomatic Achilles tendons in runners [18,19] and in a general patient population [20,21]. These findings indicate that there are pronounced differences in mechanical, morphological, and functional (i.e., muscle strength or jump performance) properties between the symptomatic and the asymptomatic Achilles tendon, which may be linked to injury etiology or progression.

Asymmetries may develop and persist (long) after being exposed to an injury. Following surgical repair after Achilles tendon rupture, tendon stiffness [22] and cross-sectional area (CSA) [23] are much larger even many years after surgery when compared to the non-operated side. In some cases, deficits in muscle mass, strength or function may even be permanent [24]. Hence, evidence on inter-limb asymmetries in patients suffering from Achilles tendinopathy or patients having been subjected to Achilles tendon rupture is well established.

Despite this, asymmetries in the healthy population are common and non-pathological to a certain extent. The inter-limb comparison of healthy Achilles tendon’s mechanical and morphological properties revealed an absolute asymmetry index ranging from 3–31% [25]. Laterality as well as specific exercise activities may contribute to inter-limb asymmetries, with increased Achilles tendon CSA and tendon thickness being reported for the dominant side in elite badminton players [26] and greater Achilles tendon stiffness and greater Young’s modulus being detected in the take-off leg of jumpers [27]. Similarly, in the patellar tendon, a greater tendon CSA in addition to increased tendon stiffness have been detected in the dominant side of fencers and badminton players [28]. Hence, asymmetries in the healthy population due to laterality and/or specific activities might be regarded as being physiological to a certain extent. Thus, there seems to be a fine line between physiological and pathological asymmetry.

It is well known that in general, exercise activity even at recreational level induces structural (i.e., material, and morphological) adaptations of the tendon which affect the mechanical properties of the structure and cause e.g., increases of tendon stiffness [29,30]. Accordingly, exercise also plays a key role in rehabilitation and therapy of tendinopathic tendons [31,32,33,34]. A common exercise therapy approach in Achilles tendinopathy is the eccentric exercise protocol developed by Alfredson [35,36]. As the adaptational response may be higher when higher tendon strains are applied [37,38,39], exercise protocols with higher load intensities, such as heavy slow resistance training [40] or the progressive loading program [41] have been applied in Achilles tendinopathy. Indeed, a high-loading exercise protocol, in which high tendon strain was induced, led to improved mechanical, and morphological tendon properties, and a greater strength of the tendinopathic side in Achilles tendinopathy patients [42]. Thus, therapeutic exercises may reduce leg asymmetries originating from Achilles tendinopathy.

To examine the effect of therapeutic exercises in Achilles tendinopathy, we analyzed an intervention trial with Achilles tendinopathy patients in which we compared eccentric exercise as gold standard with an evidence-based tendon high-loading protocol, and measured tendon mechanical, material, morphological and functional properties, tendon vascularization, GM muscle architecture, and function (i.e., muscle strength, jump performance and patient-reported outcome measures (PROM)) at baseline and after three months of intervention.

We aimed to investigate: (a) the effect of unilateral mechanical loading on inter-limb symmetry; (b) whether different loading protocols differ in their effect on inter-limb asymmetry; and (c) whether potential changes in asymmetry correlate with improved PROM (i.e., VISA-A score).

We hypothesized inter-limb asymmetry in Achilles tendinopathy at baseline in terms of lower plantar flexor muscle strength, lower tendon stiffness, higher maximum tendon strain and larger tendon CSA for the tendinopathic side. Furthermore, we anticipated that evidence-based unilateral exercise treatment would reduce aforementioned inter-limb asymmetry. We also hypothesized that a protocol with higher applied tendon strain would have a greater positive impact on inter-limb asymmetry and that a greater positive impact on asymmetry would correlate with a reduction in tendinopathy symptoms (VISA-A score).

## 2. Materials and Methods

### 2.1. Participant Characteristics and Experimental Design

This is an ancillary study of data collected in the controlled clinical trial registered under ClinicalTrials.gov (NCT02732782). In the original clinical trial, we compared the effects of different physiotherapeutic interventions on tendon mechanical and morphological properties and function in Achilles tendinopathy patients [42]. In addition, and not previously published, we measured and analyzed data from each individual’s unaffected leg to assess inter-limb symmetry as well as plantar flexor muscle architecture of both legs. Thus, all details about the process of obtaining data (i.e., eligibility criteria, allocation-to-groups, blinding, flowchart, timeline, and the adherence to the Consolidated Standards of Reporting Trials (CONSORT) Statement etc.), as well as details about the applied measurement methods for all data, except for the analysis of plantar flexor muscle architecture and the asymmetry calculation, have been thoroughly described elsewhere [42]. In the clinical trial, 44 patients were included [42]. The condition of Achilles tendinopathy was confirmed via ultrasound (at least discrete hypo-echogenic areas within the tendon) and clinical assessment by a medical doctor. In addition, a physiotherapist (G.R.) palpated the Achilles tendon for pain location to further ensure whether the reported pain is located at the tendon. Upon confirmation of the pathology and a severity level of less than 80 points on the VISA-A score, patients were assigned by sequential allocation to one of three intervention groups [42]. As we intended to assess inter-limb asymmetry, exclusion of all patients with bilateral symptoms was mandatory. Thus, in our present study, we analyzed 41 unilaterally affected Achilles tendinopathy patients for baseline inter-limb asymmetry. Out of this sample, we assessed all unilateral affected patients who underwent either the Alfredson (n = 11) or the high-load protocol (n = 13) for PRE-to POST changes in inter-limb asymmetry. The baseline characteristics of the overall patient group as well as of both intervention groups are described in Table 1. The analysis of all parameters was conducted for both the affected and the unaffected leg at baseline and three months after completion of the intervention. 

### 2.2. Intervention

The Alfredson group performed eccentric exercises with six sets of 15 repetitions twice daily according to the commonly known protocol [35]. As per protocol, eccentric-only contractions of the plantar flexors of the affected leg were performed (heel-drop) while the healthy leg performed concentric plantar flexor muscle contractions (heel-rise) to return to the start position. The high-load group performed a highly intensive unilateral strength training protocol consisting of five sets of four plantar flexor contractions at 90% of isometric maximum voluntary contractions (MVC) held for three seconds followed by three seconds of rest each and with 1-min rest in between sets with the affected leg [37,38,39,42,43]. This protocol was executed four times a week with a mobile feedback-fitted sling which has previously been described [43]. The distribution of sessions within a week was chosen individually. Load progression was allowed for both groups after the first two weeks, if pain level on the numeric rating (NRS) scale was <6/10 [44] and the individual rating of perceived exertion was <3/10 (NRS scale) [45] (Table 2). Due to ethical reasons, all patients received the option of 12 sessions of passive therapeutic treatment (i.e., manual therapy, tissue, or joint mobilization). Moreover, participants were allowed to continue with their training habits if pain levels were ≤3/10 (NRS scale) [44]. Any additional strength training of the plantar flexors or newly started lower body training was prohibited during the intervention phase. Table 2 depicts the characteristics of both exercise treatment interventions.

### 2.3. Plantar Flexor Muscle Characteristics

#### 2.3.1. Maximum Ankle Joint Moment 

The exact method for calculating the maximum ankle joint moment (plantar flexor MVC) has been described previously [37,38,39,42]. Briefly, the patients performed 2–3 maximum isometric plantar flexor contractions on a dynamometer (Biodex System 3, Biodex Medical Systems Inc., Shirley, NY, USA) with fixed ankle angle and extended knee. To take axis misalignment into account, an inverse dynamic approach was used [46] for plantar flexor moment calculation based on kinematic data from an infrared motion capture system (Vicon Nexus, version 1.7.1, Vicon Motion Systems, UK). The contribution of the antagonistic muscles to the plantar flexor moment was considered based on the m. tibialis anterior activity during the plantar flexor MVC [47] measuring using surface EMG electrodes that was related to a separately established EMG activity-dorsiflexion moment relationship (Myon m320RX, Myon AG, Luzern, Switzerland, 1000 Hz). 

#### 2.3.2. Plantar Flexor Muscle Architecture

We assessed the fascicle length, pennation angle, and thickness of the m. gastrocnemius medialis (GM) muscle using ultrasonography at 0°-degree ankle angle. A 10 cm ultrasound transducer (7.5 MHz, My Lab60, Esaote, Genova, Italy) was positioned longitudinally at ≈50% of the GM length capturing two ultrasound videos while the patients’ GM muscle remained relaxed. To prohibit any potential tilt of the probe, a custom-made plastic cast was applied. For ultrasound image analysis, a custom-made MATLAB interface (The MathWorks, version 2012, Shirley, NY, USA) was used. In 10 consecutive frames of each video, four reference points along both the upper and deeper aponeuroses were manually defined and displayed as linear-fitted lines [48]. GM muscle thickness was determined calculating the distance between the aponeuroses. A reference fascicle was generated semi-automatically by the program based on the detection of visible bits of inter-fascicular collagen bundles. Fascicle length then was defined as the length of the reference fascicle and the pennation angle as the angle between the reference fascicle and the deeper aponeurosis [49]. Average values of all the frames were used for data analysis. For proper PRE-to-POST repositioning of the ultrasound probe, we followed the previously described procedure that referenced the probe position to fixed anatomical landmarks [48,49].

### 2.4. Achilles Tendon Relative and Absolute Properties

Achilles tendon stiffness, tendon strain, tendon force and tendon elongation were analyzed using dynamometry, electromyography, motion capture and ultrasonography. These methods have been comprehensively reported elsewhere [37,38,39,42]. Briefly, the patients conducted five ramped plantar flexor MVCs within the same setting as earlier described for maximum ankle joint moment assessment (see Section 2.3.1). We calculated Achilles tendon force by dividing the maximum ankle joint moment by the tendon lever arm. The lever arm was measured using the tendon-excursion method and considering a corrective factor for the lever arm changes due to tendon alignment during contraction [50]. Thus, in contrast to the maximum ankle joint moment, the tendon force is lever-arm dependent. We calculated tendon stiffness based on the ratio of tendon force to tendon elongation between 50 and 100% of the maximum tendon force. Tendon elongation was assessed during the ramped MVCs by placing the B-mode ultrasound probe to record the GM musculotendinous junction (MTJ) displacement. Potential effects on tendon elongation measurements due to unavoidable ankle joint rotations during the MVCs were considered by an additional trial where the change of MTJ position was determined during a passive ankle joint rotation by the dynamometer at 5°/s and then applied for the ankle angle changes observed during the MVC [51]. Maximum tendon strain was calculated as the ratio of maximum tendon elongation to Achilles tendon rest length with the latter measured at 20° plantar flexion angle [52]. 

The CSA of the free Achilles tendon was assessed either with a 0.25 T magnetic resonance imaging (MRI) scanner (G-Scan, Esaote, Italy) or a 1.5 T MRI scanner (Siemens-Avanto, Siemens, Erlangen, Germany) while the same scanner was used for every PRE-POST pair. Details of the analysis have been reported previously [37,42]. Briefly, patients were positioned supine with extended hips and knees while the ankle joint was fixed at 90°. For the analysis, the transversal scans were manually segmented using the software OsiriX (Pixmeo SARL, version 2.5.1, Switzerland) [53]. Tendon CSA was manually determined and calculated in 10% increments across the whole free tendon length. Average Achilles tendon CSA was calculated as mean of all 10% increments.

The Young’s modulus of the Achilles tendon was calculated by multiplying tendon stiffness with the quotient of tendon rest length and tendon CSA. Stress of the Achilles tendon was determined as the ratio of maximum tendon force and mean Achilles tendon CSA.

The assessment of intratendinous vascularity has been comprehensively described elsewhere [42]. Briefly, vascularity was assessed in prone position with the knees extended and the ankle joint passively stabilized at 90° with relaxed plantar flexor muscles using pulsed-waved power doppler ultrasonography (7.1 MHz, My Lab60, Esaote, Genova, Italy). The 10 cm transducer was aligned parallel to the tendon visualizing the proximal portion of the calcaneal bone and the Achilles tendon within a standardized rectangle frame (10 × 2 cm) as the region of interest. Within this frame, we analyzed intratendinous vascularity solely within tendinous tissue and overall vascularization within the region of interest. Thus, overall vascularization measures consisted of intratendinous and paratendinous vascularization including Kager´s fat pat, retrocalcaneal bursa and other peritendinous tissue. Three scans with a duration of four seconds each were recorded and the frame with both highest signal activity and without any artefacts was chosen for analysis. Analysis was performed using a custom-written MATLAB script (The MathWorks, version 2012, Natick, MA, USA) quantifying the number of colored pixels (i.e., vascularization) and converting them to mm^2^.

### 2.5. Jump Performance

We assessed drop jump (DJ) and countermovement jump (CMJ) height as described previously [43]. Briefly, five maximum effort CMJs and five DJs were performed. DJs were performed from a 15 cm box. Ground reaction forces were measured with two separate force plates at a rate of 1000 Hz (Kistler, Type 9260AA, 600 × 500 × 50 mm, Winterthur, Switzerland) linked to an analogue digital converter (DAQ-System, USB 2.0, Type 5691A1). Data were recorded (BioWare Software, version number 2812A) and jump height was calculated based on the impulse momentum method [54] for the CMJ and the flight-time method [55] for the DJ, using a custom written MATLAB interface (version R2012a; MathWorks, Natick, MA, USA). For further analysis, the mean of the highest three jumps out of five attempts was used. Based on the recorded kinetic data, the impulse percentage of each leg proportional to the total impulse of every jump was calculated and reported.

### 2.6. VISA-A Score

We used the VISA-A score [56] which has been reported to be valid and reliable [57] and is commonly used as a patient reported outcome measurement to determine clinical severity of Achilles tendinopathy.

### 2.7. Leg Dominance

Leg dominance was determined by inquiry at the baseline measurement.

### 2.8. Statistics

We examined the normality of data distribution with the Kolmogorov-Smirnov-Test. Two-tailed paired *t*-tests were applied for the overall patient baseline comparison of mean values between the asymptomatic and symptomatic leg as well as for all PRE to POST AAI comparisons within an intervention group. Unpaired two-tailed *t*-tests were used for baseline characteristic comparisons between the Alfredson and high-load group, the comparison of AAI POST high-load with AAI POST Alfredson and the comparison of the Δ AAI PRE-POST between high-load and Alfredson group. AAI calculation was based on Karamanidis et al. [58] and calculated as follows:

$$Absolute\;Asymmetry\;Index\;\left(AAI\right)=\frac{\left|asymptomatic-symptomatic\right|}{0.5\;\left(asymptomatic+symptomatic\right)}\times 100\%$$

The interpretation of the Pearson correlation coefficient (r) describing the strength of the linear relationship between variables was: weak (0.0–0.3), moderate (0.3–0.5) or strong (>0.5). The relationship of the dominant to the injured side was established with an adjusted Pearson´s contingency coefficient. For all statistics, significance level was set at α = 0.05 and the software SPSS Statistics (IBM, version 21, Armonk, NY, USA) was used. 

## 3. Results

### 3.1. Baseline Comparison between the Symptomatic and the Asymptomatic Side

In the overall patient group (n = 41) the symptomatic leg was characterized by a significant lower tendon force (*p* = 0.017), a larger mean CSA (*p* < 0.001), lower tendon stress (*p* < 0.001), more pronounced intratendinous (*p* = 0.042) and overall vascularization (*p* = 0.021) in comparison to the asymptomatic leg (Table 3). No significant difference between the asymptomatic and symptomatic leg was detected for maximum plantar flexor moment (*p* = 0.967), GM muscle fascicle length (*p* = 0.521), GM pennation angle (*p* = 0.463), GM muscle thickness (*p* = 0.901), Achilles tendon maximum elongation (*p* = 0.617), tendon stiffness (*p* = 0.429), maximum tendon strain (*p* = 0.590), Youngs modulus (*p* = 0.294), CMJ (*p* = 0.065) and DJ impulse percent of total (*p* = 0.254). 

#### 3.1.1. Cross-Sectional Area

**Figure 1:**
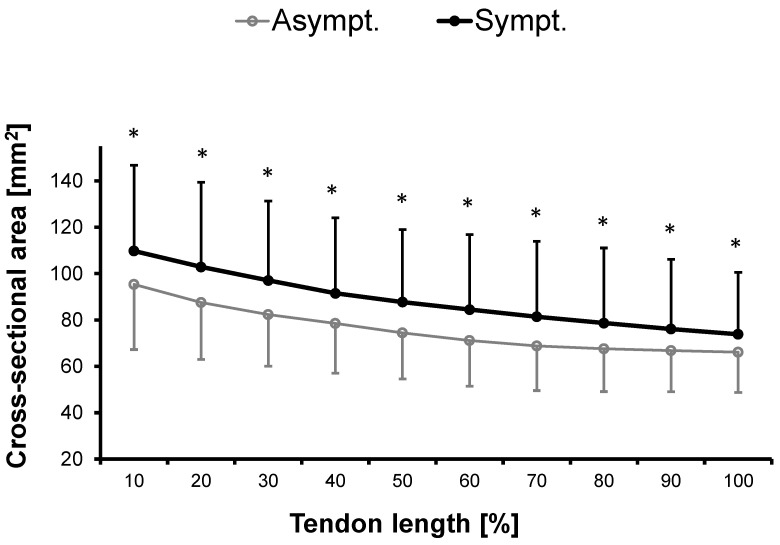
Cross-sectional area (CSA) (mm^2^) of the asymptomatic and symptomatic Achilles tendon of the overall patient group at baseline (n = 36). The CSA was measured in 10% steps of tendon length from distal (0%) to proximal (100%) alongside the free Achilles tendon. * indicates significant difference when compared to the corresponding tendon region between legs (*p* < 0.05).

The Achilles tendon CSA was significantly larger in the symptomatic compared to the asymptomatic leg along the entire length of the free tendon measured at 10% intervals (Figure 1). The mean difference between legs ± SD and the corresponding *p*-values for the 10% intervals were as follows: 0–10%: 14.4 ± 27.8 mm^2^ (*p* = 0.005), 10–20%: 15.3 ± 26.7 mm^2^ (*p* = 0.002), 20–30%: 14.7 ± 24.4 mm^2^ (*p* = 0.001), 30–40%: 13.0 ± 24.3 mm^2^ (*p* = 0.002), 40–50%: 13.3 ± 20.4 mm^2^ (*p* = 0.001), 50–60%: 13.3 ± 20.6 mm^2^ (*p* = 0.001), 60–70%: 12.6 ± 20.2 mm^2^ (*p* = 0.001), 70–80%: 11.1 ± 19.8 mm^2^ (*p* = 0.003), 80–90%: 9.3 ± 18.2 mm^2^ (*p* = 0.006) and 90–100%: 7.7 ± 16.3 mm^2^ (*p* = 0.009).

#### 3.1.2. Laterality

In the overall patient group, leg dominance was not related to the injured side according to the corrected Pearson’s contingency coefficient C_korr_ = 0.049 (*p* = 0.546).

### 3.2. Absolute ASYMMETRY INdex

The AAI PRE (i.e., AAI between symptomatic and asymptomatic leg of the overall patient group at baseline) ranged from 9–91% with the highest asymmetries recorded for intratendinous vascularization and the lowest asymmetries for muscle fascicle length (Table 3). 

**Figure 2:**
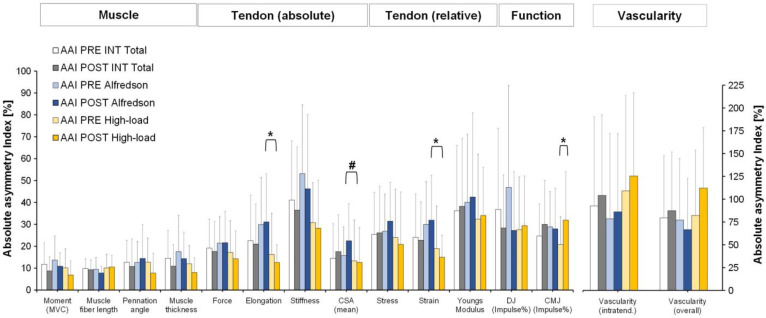
Absolute asymmetry index (AAI) for muscular, tendinous (absolute), tendinous (relative) and functional (i.e., jump performance) parameters of the plantar flexor muscle-tendon unit and for Achilles tendon vascularity for both intervention groups at baseline (AAI PRE INT TOTAL) (n = 24) and post exercise (AAI POST EXP) (n = 24), PRE (AAI PRE high-load) and post 12 weeks of specific high-loading exercise (AAI POST high-load) (n = 13) and PRE (AAI PRE Alfredson) and post 12 weeks of specific eccentric exercise (AAI POST Alfredson) (n = 11). Data are presented as mean ± standard deviation (SD). MVC = maximum voluntary isometric plantar flexor muscle contraction; CSA = cross-sectional area of Achilles tendon; intratend. = intratendinous; overall = intratendinous and paratendinous vascularization; CMJ = counter movement jump; DJ = drop jump; Impulse % = impulse percent of total; * significant difference between AAI (*p* < 0.05); # significant difference between comparisons of AAI PRE to POST differences (*p* < 0.05).

Comparisons between AAI PRE INT Total (INT TOTAL = both interventions) and AAI POST INT Total (i.e., AAI between asymptomatic and symptomatic leg after 12 weeks of unilateral exercise for the injured leg) revealed no significant differences for any parameter (Figure 2).

Comparisons between AAI PRE Alfredson and AAI POST Alfredson (i.e., AAI between asymptomatic and symptomatic leg after 12 weeks of specific eccentric unilateral exercise for the injured leg) revealed no significant differences for any of the parameters (Figure 2).

Comparisons between AAI PRE high-load and AAI POST high-load (i.e., AAI between asymptomatic and symptomatic leg after 12 weeks of specific high-loading unilateral exercise for the injured leg) revealed significant differences for the CMJ (impulse percent of total) (*p* = 0.034) (Figure 2) increasing by 11.12 ± 15.87 from PRE to POST.

Comparisons between intervention groups revealed significant differences between AAI POST high-load and AAI POST Alfredson for tendon elongation (*p* = 0.021) and tendon strain (*p* = 0.026) (Figure 2) indicating lower AAIs in the high-load group.

Comparisons of the AAI differences from PRE to POST between the high-load and the Alfredson group revealed a significant difference for the tendon CSA (*p* = 0.045) with an AAI PRE to POST decrease of −0.6 ± 5.3 for the high-load group and an AAI PRE to POST increase of 6.7 ± 10.9 for the Alfredson group (Figure 2).

### 3.3. Correlation to VISA-A Score

In the overall intervention group, the change in VISA-A score from PRE to POST did not correlate with PRE to POST changes in the AAI in any parameter. 

**Figure 3:**
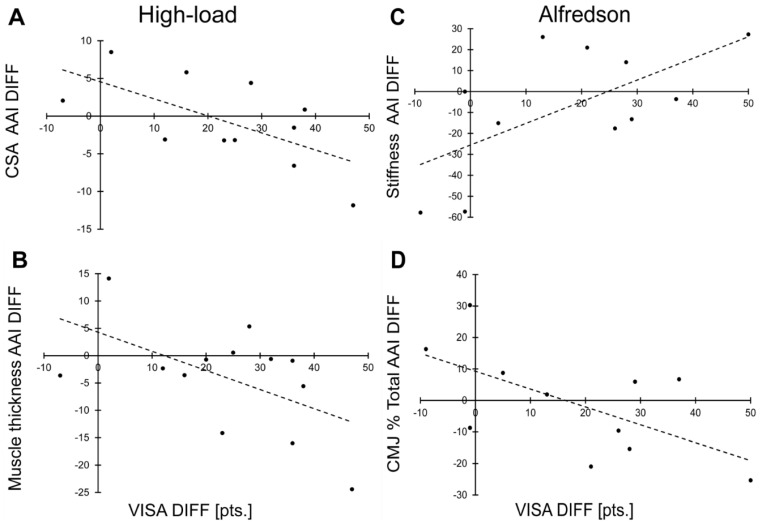
Correlations between the Victorian Institute of Sport Assessment-Achilles questionnaire (VISA-A) change from PRE to POST and the absolute asymmetry index (AAI) change from PRE (at baseline) to POST (after 12 weeks of exercise) in the high-load group for Achilles tendon cross-sectional area (CSA) (**A**) and plantar-flexor muscle thickness (**B**) and in the Alfredson group for Achilles tendon stiffness (**C**) and the percent impulse ratio of both legs for the counter movement jump (CMJ) (**D**), DIFF = difference, pts. = points.

Within the high-load group, PRE to POST improvements in the VISA-A score correlated significantly with CSA AAI reductions (r = −0.620; *p* = 0.024) and by trend with muscle thickness AAI reductions (r = −0.545; *p* = 0.054) (Figure 3A,B).

Within the Alfredson group, PRE to POST improvements in the VISA-A score correlated significantly with CMJ percent impulse ratio AAI reductions (r = −0.616, *p* = 0.044) and a tendon stiffness AAI increase (r = 0.633; *p* = 0.037) (Figure 3C,D). 

## 4. Discussion

In agreement with the literature and with our hypothesis, we found inter-limb asymmetries in Achilles tendinopathy with AAIs ranging from 9–91% for mechanical, material, morphological and functional Achilles tendon parameters. When comparing the tendinopathic with the asymptomatic side, the tendinopathic tendon was characterized by significant lower tendon force, lower tendon stress, larger mean tendon CSA as well as increased intratendinous and tendon overall vascularization. The tendon CSA was larger along the whole length of the free tendinopathic Achilles tendon. In contrast to our hypothesis, exercise therapy did not in general lead to a reduction in asymmetries. While intervention-dependent effects were detected, it cannot clearly be confirmed that higher applied tendon strain has a greater positive impact on inter-limb asymmetry, as e.g., asymmetry increased with therapy in one parameter in the high-load group. Lastly, as hypothesized, reductions in asymmetry correlated with reductions in tendinopathy symptoms (VISA-A score increase) for the high-load group regarding tendon CSA and for the Alfredson group in CMJ percent impulse ratio. However, in contrast to our hypothesis, a VISA-A improvement was also associated with an AAI increase of tendon stiffness in the Alfredson group.

While a certain degree of limb asymmetry can be considered as non-pathological, pronounced inter-limb differences particularly in Achilles tendon CSA and vascularization appear to be a hallmark of Achilles tendinopathy. Enlarged tendinopathic Achilles tendons have been reported in many studies [18,19,20,21,59,60,61] and may be caused by inflammation related edema. Localized fusiform tendon swelling has been reported previously in mid-portion Achilles tendinopathy [61]. However, our findings indicate that the increase in tendon CSA was not restricted to a specific location within the tendon but was apparent throughout the entire length of the free Achilles tendon. Although leg dominance can affect inter-limb tendon CSA asymmetry, laterality was not related to the side of injury in our study, making it unlikely that the larger CSA was caused by more intense loading. Furthermore, there is compelling evidence confirming our results of more pronounced tendon vascularity in tendinopathic Achilles tendon [18,19,20,21]. Indeed, vascular alterations in tendinopathy are well known [62] and are reported to correlate with severity [34], although a considerable proportion of tendinopathy patients (i.e., 37%) does not show any sign of neovascularization [63], questioning increased vascularization as a single valid clinical sign for all patients [64]. Both increased tendon CSA and vascularization may have been a result of inflammatory processes following tendon injury. Thus, asymmetries in those two parameters are most likely caused by tendinopathy and not risk factors predisposing to tendinopathy. 

In contrast, tendon mechanical properties such as reduced stiffness or increased strain have been discussed to be a risk factor for tendinopathy [65]. Current research assessing inter-limb asymmetries for mechanical tendon properties in Achilles tendinopathy reported reduced tendon strain [66] and reduced tendon stiffness [17,67,68,69]. Additionally, based on comparisons to matched controls, lower tendon stiffness and higher tendon strain have been reported in Achilles tendinopathy [59,70]. Shear wave elastography measurements confirmed findings of reduced tendon stiffness [71], leading to the idea of stiffness measures as a potential indicator for tendinopathic changes in a recent review [70]. In line with that, our results showed relatively high AAIs for Achilles tendon stiffness and tendon strain with 32.35 ± 27.22 and 22.60 ± 19.24 although it remains unclear, whether these asymmetries were the cause of or were caused by tendinopathy. Nevertheless, we did not detect any significant inter-limb tendon stiffness nor tendon strain differences. In addition, the inter-limb AAIs ranging from 9–34.6% (except tendon vascularization) were of similar magnitude as inter-limb AAIs described for healthy Achilles tendon with values ranging from 3–31% [25]. Thus, except for tendon vascularization, a pronounced limb asymmetry cannot necessarily be interpreted as pathological.

In turn, a reduction in asymmetry cannot necessarily be interpreted as being healthier. Although both types of exercise intervention reduced tendinopathic symptoms (VISA-A score increase), an improvement in tendon health was significantly associated with a reduction in asymmetry in two variables only. Those two variables differed between groups (CSA AAI reduction in the high-load group and CMJ impulse difference AAI in the Alfredson group) and are thus not representative for a general effect. Furthermore, the CMJ impulse asymmetry increased post intervention in the high-load group, and in the Alfredson group an increase in tendon stiffness asymmetry was associated with improved tendon health (VISA-A score). Those findings indicate, that within the therapeutic exercise interventions increases as well as reductions in asymmetries can occur at the same time.

It is furthermore conceivable that unilateral exercises may even provoke asymmetries or amplify existing asymmetries. The capacity of tendinous and tendinopathic tissue to adapt to mechanical loading (i.e., exercise) is well known [37,38,39,42,72,73]. We have previously reported that three months of unilateral high-loading exercise in Achilles tendinopathy patients increased tendon stiffness and CSA in the trained leg [42]. The AAI increase in inter-limb ratio of CMJ height after three months of high-loading exercises may thus suggest that a unilateral stronger, stiffer, and thicker Achilles tendon translates to less inter-limb balanced bilateral jumping maneuver. 

In healthy tendons, it is well established that high-loading exercise protocols inducing larger tendon strains result in higher increases in mechanical and morphological tendon properties than low-strain protocols [37,38,39,73]. This was recently confirmed in Achilles tendinopathy patients [42]. In this regard, POST-to-POST differences between the high-load and Alfredson group showing lower AAIs for the high-load group in tendon strain and tendon elongation are in line with the literature at least to the extent that an impact on tendon strain and thus tendon elongation might be expected. As a result, a more equilibrated tendon strain between limbs might protect the tendinopathic tendon against strain-induced re-injury. Moreover, our comparisons of the PRE to POST differences between both groups showed a decrease for the AAI of tendon CSA in the high-load group indicating a more equilibrated tendon CSA between limbs when compared to the Alfredson group that resulted in an increased tendon CSA AAI.

In this regard, it is notable that Alfredson’s eccentric exercise protocol leads to different loading of both limbs, as every repetition consists of a concentric plantar flexion (heel rise) of the unaffected limb and an eccentric dorsal flexion (heel drop) of the affected limb. This bilateral training may have a different impact on asymmetry when compared to purely unilateral exercises and may have the potential to reduce inter-limb asymmetry in bilateral movements like the CMJ. While the bilateral nature of the exercise may explain why VISA-A improvements correlated with an AAI reduction of CMJ percent impulse ratio in this group, the eccentric and concentric exercises may be associated with differences in tendon loading which may have caused the AAI increase of tendon stiffness detected in this group.

### Limitations

It might be questionable, if the status of the unaffected limb should be regarded as “normal” since evidence suggests motor control alterations to occur in both limbs [74]. In previous studies, matched-control comparisons between patients suffering from Achilles tendinopathy and healthy controls showed greater tendon CSA, lower tendon stiffness, lower Young’s modulus [59] and longer free Achilles tendon length [75] for the tendinopathic tendon. As both limbs might have been affected, comparisons with matched healthy controls may have provided further insights [74]. 

As this study has been conducted with male patients only the transfer of the results to female patients is limited, particularly as we know that symptoms and regenerative processes may differ between sexes [44,76,77]. Thus, it is necessary to acquire additional data from female patients, to be able to consider sex differences in therapeutic approaches or to make recommendations for the general population.

Without follow-up assessments of the tendon properties, our study design does not allow to adequately assess long-term effects. Future research with follow-up assessments in addition to a longer monitoring period may identify potential long-term effects empirically.

## 5. Conclusions

In Achilles tendinopathy the symptomatic side significantly differs from the asymptomatic contralateral limb. However, the role and meaning of asymmetry regarding tendon health is still unclear as, except for tendon vascularity, the AAIs are in the same range as previously seen in the healthy population. Furthermore, therapeutic exercise interventions have little effect in terms of pronounced asymmetry reductions in Achilles tendinopathy, highlighting the difficulty to use the comparison with the contralateral limb to evaluate therapeutic progression. While specific unilateral or bilateral exercise interventions may have small but differing effects on asymmetries, a reduction in asymmetry is not necessarily associated with an improvement in tendon health while an increase in asymmetry does not necessarily indicate a reduction in health. To inform clinical practice, decisions regarding return-to-sport should neither exclusively be based on the presence nor the reduction of inter-limb asymmetry. In this regard, it might be advantageous to acquire baseline pre-injury data for both limbs in athletes which can be used as reference in case of injury. Future research might further investigate the fine line between physiological and pathological asymmetry to establish a more profound understanding of relative inter-limb asymmetry changes and their clinical implications.

## Figures and Tables

**Table 1:** Baseline characteristics of overall patient group (n = 41) and of intervention groups: Alfredson group (n = 11) and high-load group (n = 13). Values of baseline characteristics are presented as mean ± SD (standard deviation) and range in parentheses.

Baseline Characteristics	All Patients (n = 41)	Alfredson (n = 11)	High-Load (n = 13)
Age [years]	40.2 ± 9.2 (24–55)	38.7 ± 8.8 (24–52)	40.1 ± 8.4 (27–52)
Body height [cm]	181.9 ± 5.8 (173–198)	180.0 ± 4.8 (175–186)	184.1 ± 6.4 (177–198)
Body mass [kg]	81.4 ± 10.6 (64–110)	77.5 ± 7.4 (65–88)	86.1 ± 11.7 (70–110) *
Body mass index [kg/m^2^]	24.6 ± 3.0 (20–37)	23.9 ± 2.0 (21–28)	25.3 ± 2.9 (22–32)
Activity level [hours/week] ^A^	6.7 ± 4.6 (0–22.5)	5.9 ± 4.0 (1–12)	8.2 ± 5.4 (2–22.5)
Laterality (left/right)	6/32 ^A^	1/9 ^B^	2/11
Symptomatic AT (left/right)	24/17	4/7	7/6
Symptom localization (Ins./mid-portion)	17/24	6/5	6/7
Symptom duration [months]	25.9 ± 45.7 (3–264)	44.5 ± 77.6 (3–264)	21.2 ± 31.2 (3–96)
VISA-A PRE [points]	57.4 ± 13.9 (19–78)	41.1 ± 14.8 (29–73)	58.4 ± 11.7 (36–73)

^A^ n = 38; ^B^ n = 10; AT = Achilles tendon; Ins. = insertional; VISA-A PRE = Victorian Institute for Sports Assessment—Achilles tendon score at baseline. * significant difference to Alfredson group (*p* = 0.047).

**Table 2:** Characteristics of both therapeutic interventions: Eccentric exercises (i.e., the Alfredson protocol) and high-loading exercises.

	Alfredson	High-Load
Procedure	Upright standing position, exercises were carried out both with extended knee and bended knee. Start position: Only the forefoot of the injured leg was placed on the edge of a stair at maximum plantar flexion, lowering the heel under full body weight with an eccentric phase of 3 s. We ensured eccentric-only contractions of the plantar flexors by using the healthy leg to return to the start position. The use of a full ankle range of motion in the eccentric phase was encouraged.	Seated position on the floor with extended knees and a feedback-fitted sling fixed around the pelvis that was equipped with a foot plate [43]. The individual training load was calculated based on 90% of the mean of five executed MVCs. Start position: The forefoot (with shoes) was placed in the foot plate. The sling was adjusted as tightly as possible, to execute 90% isometric plantar flexor contractions at a standardized ankle angle position (90°) (i.e., foot sole 90° perpendicular to the tibia).
Warm-up	No warm-up.	Three sets of five isometric sub-maximal plantar flexor contractions with a rest of1 min in between.
Repetitions per session	Three sets of 15 reps. with extended knee and three sets of 15 reps. with bended knee.	Five sets of four repetitions, each contraction lasting 3 s at 90% isometric plantar flexor MVC, followed by 3 s rest.
Resting time	One min after each set.	One min after each set.
Frequency	Two sessions per day.	Four times per week.
Pain	Up to 5/10 (NRS scale for pain) was allowed for exercise treatment, up to 3/10 (NRS scale for pain) for other activities [44].	Up to 5/10 (NRS scale for pain) was allowed for exercise treatment, up to 3/10 (NRS scale for pain) for other activities [44].
Optional progression	Up to 5% load increase per week; progression was allowed after 2 weeks and only if pain level was < 6/10 (NRS scale) [44] and individual rating of perceived exertion was <3/10 (NRS scale) [45].	Up to 5% load increase per week; progression was allowed after 2 weeks and only if pain level was <6/10 (NRS scale) [44] and individual rating of perceived exertion was <3/10 (NRS scale) [45].

MVC = maximum voluntary contraction; NRS = numeric rating scale; reps. = repetitions.

**Table 3:** Comparison of Achilles tendon mechanical, morphological, material, and functional properties, as well as vascularization of the plantar flexor muscle-tendon unit between asymptomatic and symptomatic leg and corresponding absolute asymmetry indices (AAI) of the overall patient group at baseline (PRE). Data are presented as mean ± standard deviation (SD). The corresponding pearson correlation coefficient (r) between sides is reported.

Parameter	Asymptomatic PRE (n = 41)	Symptomatic PRE (n = 41)	r	AAI PRE (n = 41)
Moment (MVC) [Nm]	239.61 ± 44.30	239.41 ± 41.65	0.729 ^#^	9.71 ± 8.77
Force [N]	3642.90 ± 824.51	3411.42 ± 759.79 *	0.715 ^#^	14.91 ± 11.99
Muscle fiber length	73.66 ± 11.51	74.45 ± 11.44	0.769 ^#^	9.07 ± 5.64
Pennation angle	16.41 ± 2.45	16.15 ± 2.00	0.488 ^#^	11.86 ± 8.66
Muscle thickness	18.84 ± 2.91	18.90 ± 3.18	0.542 ^#^	11.55 ± 11.52
Elongation [mm]	12.29 ± 2.72	12.04 ± 3.39	0.454 ^#^	21.29 ± 20.22
Stiffness [N/mm]	358.46 ± 123.05	378.50 ± 112.59	0.074	32.35 ± 27.22
Strain [%]	6.22 ± 1.42	6.07 ± 1.75	0.425 ^#^	22.60 ± 19.24
CSA (mean) [mm^2^]	76.44 ± 19.38 ^A^	87.42 ± 28.62 ^A,^*	0.798 ^#^	14.89 ± 14.19 ^A^
Youngs modulus [GPa]	0.99 ± 0.38 ^A^	0.92 ± 0.34 ^A^	0.358 ^#^	34.58 ± 26.34 ^A^
Stress [MPa]	50.10 ± 16.94 ^A^	42.17 ± 16.18 ^A,^*	0.826 ^#^	23.83 ± 17.25 ^A^
Vasc. (intratend.) [mm^2^]	1.51 ± 3.26	5.15 ± 11.15 *	0.213	90.70 ± 94.86
Vasc. (overall) [mm^2^]	10.75 ± 8.80	16.74 ± 16.21 *	0.292	84.94 ± 60.35
CMJ (Impulse %) [%]	52.33 ±7.68 ^B^	47.66 ± 7.68 ^B^	n. a.	27.41 ± 16.19 ^B^
DJ (Impulse %) [%]	52.13 ± 11.50 ^B^	47.86 ± 11.50 ^B^	n. a.	33.24 ± 32.53 ^B^

^A^ n = 36; ^B^ n = 39; * significant difference to asymptomatic (*p* < 0.05); ^#^ statistically significant Pearson correlation coefficient; n. a. = not applicable; AAI = absolute asymmetry index; AAI PRE = AAI between symptomatic and asymptomatic leg at baseline; MVC = maximum voluntary isometric plantar flexor muscle contraction; CSA (mean) = averaged cross-sectional area of Achilles tendon; Vasc. = vascularity; intratend. = intratendinous; overall = intratendinous and paratendinous vascularization; Impulse % = impulse percent of total impulse; CMJ = counter movement jump; DJ = drop jump.

## Data Availability

The datasets generated during and/or analyzed during the current study are available from the corresponding author on reasonable request.
